# Dynamic Analyses of the Short‐Term Effects of Different Bisphosphonates Using Intravital Two‐Photon Microscopy

**DOI:** 10.1002/jbm4.10057

**Published:** 2018-06-22

**Authors:** Junichi Kikuta, Mai Shirazaki, Takao Sudo, Hiroki Mizuno, Akito Morimoto, Riko Suehara, Masafumi Minoshima, Kazuya Kikuchi, Masaru Ishii

**Affiliations:** ^1^ Department of Immunology and Cell Biology Graduate School of Medicine and Frontier Biosciences Osaka University 2‐2 Yamada‐oka Suita Osaka 565‐0871 Japan; ^2^ Department of Material and Life Science Graduate School of Engineering Osaka University 2‐1 Yamada‐oka Suita Osaka 565‐0871 Japan

## Abstract

Bisphosphonates are commonly used for the treatment of bone disorders such as osteoporosis; however, the mechanism by which they affect the dynamics of living mature osteoclasts in vivo remains unknown. Here, we describe the short‐term effects of different bisphosphonates on controlling the bone resorptive activity of mature osteoclasts in living bone tissues of mice using intravital two‐photon microscopy with a pH‐sensing chemical fluorescent probe. Three types of nitrogen‐containing bisphosphonates, risedronate, alendronate, and minodronate, inhibited osteoclastic acidification during osteoporotic conditions just 12 hours after i.v. injection. Among the three types of drugs, risedronate was the most effective at increasing osteoclast motility and changing the localization of proton pumps, which led to an inhibition of bone resorption. Together, these results demonstrate that the intravital imaging system is a useful tool for evaluating the similarities and differences in currently used antibone resorptive drugs. © 2018 The Authors. *JBMR Plus* published by Wiley Periodicals, Inc. on behalf of American Society for Bone and Mineral Research.

## Introduction

Bisphosphonates (BPs) inhibit the bone resorption of osteoclasts and are widely used for the treatment of bone‐resorptive diseases, including osteoporosis, Paget disease, and metastatic cancer.^1^ More potent nitrogen‐containing BPs (N‐BPs), such as risedronate, alendronate, and minodronate, have recently been synthesized and have been shown to reduce the incidence of fractures in clinical trials. N‐BPs strongly bind to the surface of the bone mineral, hydroxyapatite (HAP), and act by inhibiting the enzyme, farnesyl pyrophosphate synthase (FPPS).^2^, ^3^ BPs with different binding affinities differ in their diffusion within the bone and uptake by osteoclasts, resulting in different therapeutic actions in vivo.^4^ These properties affect the potency and side effects of the drug in clinical use; therefore, they are of great interest to researchers and clinicians.

The exact localization of BPs within bones, as well as the ability of osteoclasts to take up BPs, has been well‐studied. Previous studies using radioactive isotopes reported that radiolabeled BPs localized to bone tissues and were taken up by osteoclasts within 24 hours after treatment in animal models.^5^, ^6^, ^7^ Another study using a fluorescent BP analogue also reported the localization of BPs on the bone surface and its cellular uptake by osteoclasts.^8^, ^9^ However, it is still unknown when BPs actually affect the dynamics of mature osteoclasts and inhibit bone resorption in vivo.

We recently established a new system for visualizing fluorescently labeled mature osteoclasts in intact bone tissues using intravital two‐photon microscopy and identified different functional subsets of living mature osteoclasts from “static‐bone resorptive (R)” to “moving‐nonresorptive (N).”^10^ Using this system, we found that the application of risedronate for 5 days reduced the total number of mature osteoclasts tightly attached to the bone surface, with many of the remaining cells representing an increase in the N population. We also developed a pH‐sensing chemical probe, pHocas‐3, which emits green fluorescence in acidic environments and enables the visualization of the local site of bone resorption in real time.^11^

In this study, using an intravital two‐photon imaging system to visualize the in vivo behavior of mature osteoclasts, we analyzed the time‐dependent effects of N‐BPs on osteoclast dynamics in vivo and showed that they change osteoclast morphology and inhibit bone resorption in living bone tissues within a short period after administration.

## Materials and Methods

### Mice

TRAP‐tdTomato mice (C57BL/6 background) and V‐type H^+^ ATPase a3 subunit‐green fluorescent protein (GFP) fusion knock‐in mice (a3‐GFP, C57BL/6 background) were previously described.^10^, ^12^ Female mice (8 weeks of age) were used in all experiments. Mice were housed at a maximum of three mice per cage and were randomly chosen for each experiment. All of the mice were fed a normal diet (Oriental Yeast Co., Ltd., Tokyo, Japan; MF) and maintained at 23°C ± 1.5°C and 45% ± 15% humidity with a 12‐hour light/dark cycle in the specific pathogen‐free animal facility of Osaka University (Osaka, Japan). All animal experiments were performed according to institutional animal experimental guidelines under approved protocols from the Animal Experimental Committee of Osaka University.

### Two‐photon intravital bone tissue imaging

Mice were anesthetized using isoflurane (Wako Pure Chemical Industries, Ltd., Tokyo, Japan). The frontoparietal region of the skull bone was exposed, and the internal surfaces of bones were observed using two‐photon microscopy.^10^ The imaging system was composed of a Nikon upright two‐photon microscope (A1R‐MP) equipped with a 25× water‐immersion objective (APO, N.A. 1.1; Nikon, Tokyo, Japan) and a Carl Zeiss upright two‐photon microscope (LSM 780 NLO) equipped with a 20× water immersion objective (W Plan‐Apochromat, N.A. 1.0; Carl Zeiss, Oberkochen, Germany). Both systems were driven by a laser (Chameleon Vision II Ti:Sapphire; Coherent, Santa Clara, CA, USA). Intravital bone imaging experiments for TRAP‐tdTomato mice were performed using a Zeiss two‐photon microscope; spectral images were acquired by specialized internal multi‐photomultiplier detectors. Acquired raw images were subjected to spectral unmixing with ZEN software (Carl Zeiss) to create unmixed images that excluded autofluorescence. The excitation wavelength of 940 nm was used. Experiments for a3‐GFP mice were performed using a Nikon two‐photon microscope, and fluorescent images were acquired by external non‐de‐scanned detectors equipped with a bandpass emission filter at 500/50 nm (for GFP). The excitation wavelength was 930 nm.

### Drug treatments

Risedronate (50 µg/kg; EA Pharma Co., Ltd., Tokyo, Japan), alendronate (100 µg/kg; Wako Pure Chemical Industries), or minodronate (20 µg/kg; Chengdu‐D‐Innovation Pharmaceutical, Chengdu, China) dissolved in PBS was administered by i.v. injection to TRAP‐tdTomato or a3‐GFP mice, and images were acquired consecutively. In the experiments for TRAP‐tdTomato mice, mice were ovariectomized 1 month prior to imaging. In the experiments for a3‐GFP mice, GST‐RANKL (Oriental Yeast Co., Ltd.; 1 mg/kg in PBS) was injected intraperitoneally into mice every day beginning 2 days prior to imaging. No adverse events were observed.

### Image analysis to track morphological changes in mature osteoclasts

Cell shapes were recognized by the image analysis software, NIS‐elements (Nikon), and three distinct areas were defined: the initial time frame (*t* = 0) (A), the final time frame (*t* = 5) (C), and the overlap between the two time frames (B). The cell deformation index, which represents the ratio of areas changed during 5 min divided by that of the previous time frame, was calculated as (A + C)/(A + B), and was described in detail previously.^10^

### Image analysis of the bone‐resorbing activity of mature osteoclasts

A pH‐sensing chemical probe (pHocas‐3) dissolved in PBS was injected subcutaneously at 7 mg/kg body weight daily into TRAP‐tdTomato mice, commencing 3 days prior to imaging.^11^ The bone‐resorbing ability of osteoclasts was assessed after image acquisition. Osteoclast areas were binarized and automatically extracted from the original images. The mean pHocas‐3 fluorescence intensities in osteoclast areas (pHocas‐3 signals) and outside these areas (pHocas‐3 noise) were measured. The bone‐resorbing index was calculated as the ratio of the pHocas‐3 signal to pHocas‐3 noise.

### Statistical analysis

All of the data were analyzed using GraphPad Prism software (GraphPad, San Diego, CA, USA) and are presented as the mean ± SD. Two‐tailed *t* tests were used to calculate *p* values. A *p* value <0.05 was considered statistically significant.

## Results

### BPs inhibited osteoclastic acidification within 12 hours after treatment

**Figure 1:**
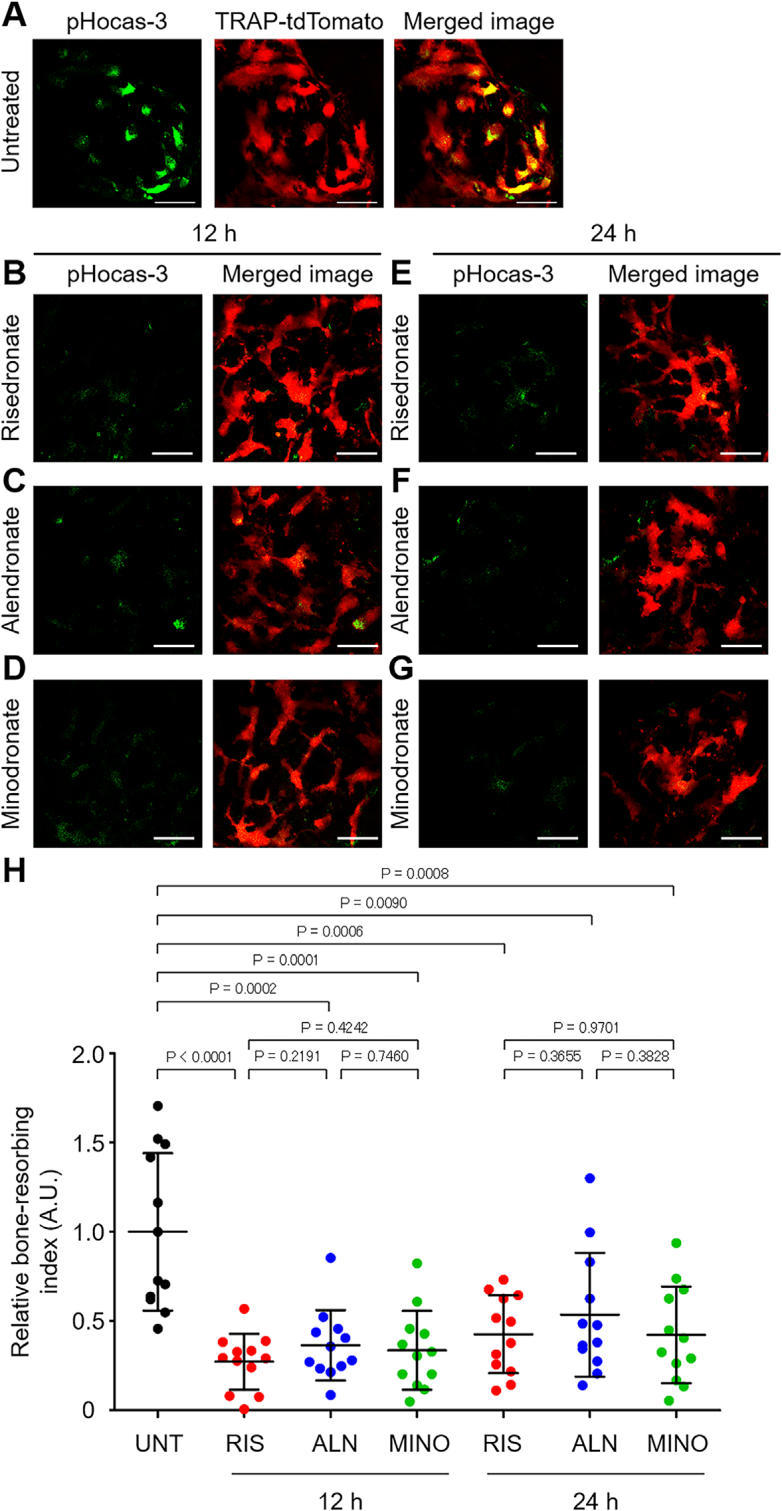
Effects of a single injection of bisphosphonates on osteoclastic acidification. (*A*) Representative images of bone resorption activity in osteoporotic TRAP‐tdTomato mice treated with the pH‐sensing chemical probe (pHocas‐3). Green fluorescent signals from a high H^+^ concentration (left), mature osteoclasts (mOCs) expressing TRAP‐tdTomato signals (middle), and merged images (right). Scale bar: 50 µm. (*B–D*) Representative images of bone resorption activity in osteoporotic TRAP‐tdTomato mice treated with pHocas‐3 at 12 hours after single i.v. administration of risedronate (*B*), alendronate (*C*), or minodronate (*D*). Green, fluorescent signals from pHocas‐3; red, mOCs expressing TRAP‐tdTomato. Scale bar: 50 µm. (*E–G*) Representative images of bone resorption activity in osteoporotic TRAP‐tdTomato mice treated with pHocas‐3 at 24 hours after single i.v. injection of risedronate (*E*), alendronate (*F*), or minodronate (*G*). Green, fluorescent signals from pHocas‐3; red, mOCs expressing TRAP‐tdTomato. Scale bar: 50 µm. (*H*) Bone resorption index of mature osteoclasts during osteoporotic conditions at 12 and 24 hours after the administration of risedronate, alendronate, or minodronate. Images were obtained from three independent experiments per group. Data are presented as the mean ± SD. UNT = untreated; RIS = risedronate; ALN = alendronate; MINO = minodronate.

First, we examined the short‐term effects of a single injection of N‐BPs on the acidification by bone‐resorbing osteoclasts in living bone tissues using intravital two‐photon microscopy with a pH‐sensing chemical fluorescent probe, pHocas‐3, to detect local low pH in bone resorption areas on the bone surface in vivo.^11^ This pH probe was injected subcutaneously into the osteoporotic mice in which mature osteoclasts were labeled with a red fluorescent protein (tdTomato). During osteoporosis, green fluorescent signals from the pH probes overlapped with most of the mature osteoclasts, suggesting that these cells were actively secreting protons and resorbing bone tissues in vivo (Fig. 1 *A* and Supplementary Video 1). Next, risedronate, alendronate, or minodronate was i.v. injected into the pH probe‐treated mice, and bone tissues were observed 12 and 24 hours later. Compared to untreated mice, at 12 hours after treatment with risedronate, alendronate, or minodronate, most of the mature osteoclasts did not overlap with green dots, which represents a low pH, and the bone‐resorbing index levels were significantly decreased (Figs. 1 *B, C, D*, *H* and Supplementary Video 1). In addition, this effect was maintained for 24 hours after i.v. injection (Figs. 1 *E, F, G, H*). These results indicate that N‐BPs inhibited proton secretion by mature osteoclasts on the surface of living bone tissues within a short period after treatment.

### BPs changed osteoclast motility within 12 hours after treatment

**Figure 2:**
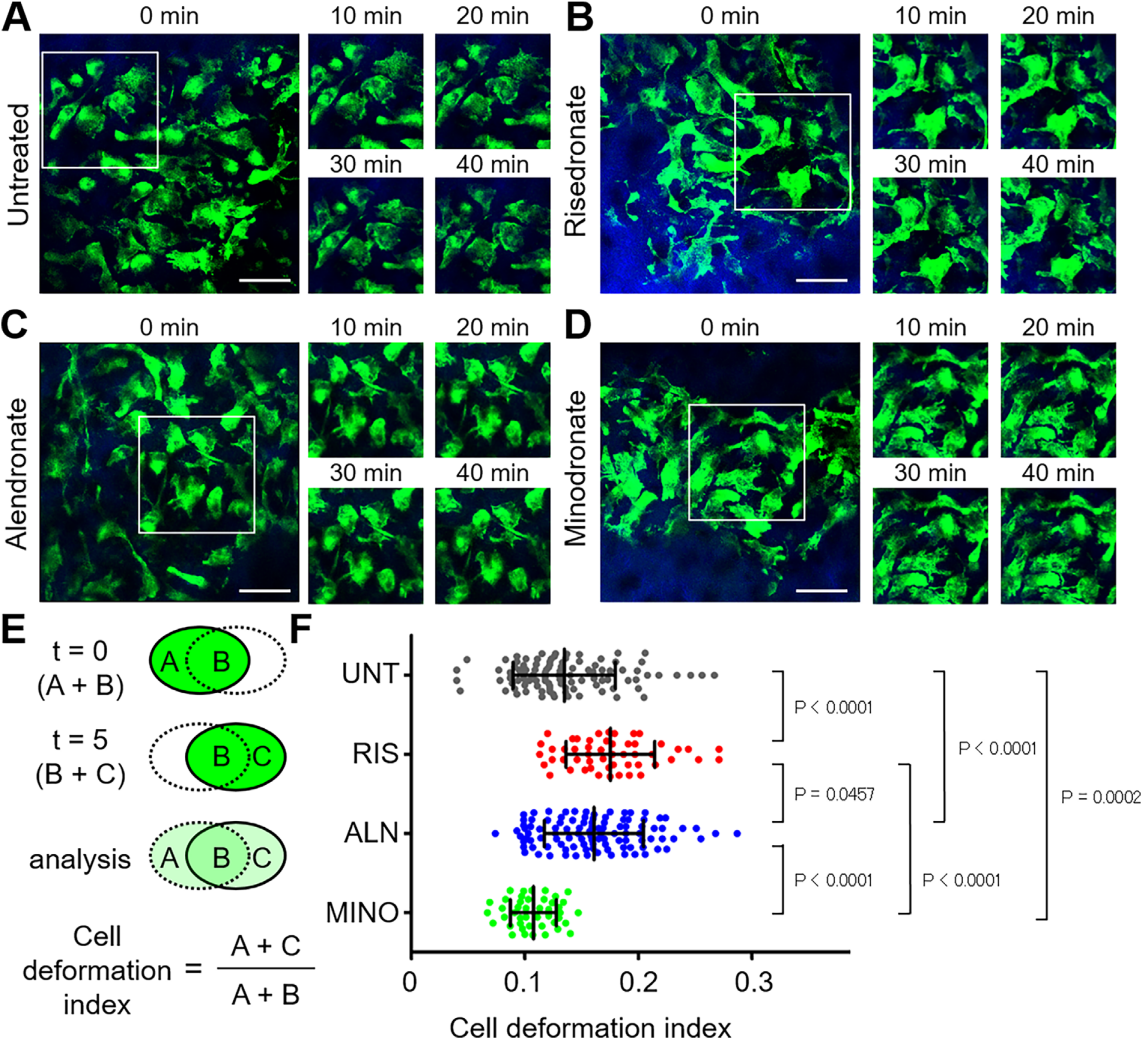
Effects of bisphosphonates on osteoclast motility at 12 hours after treatment. (*A–D*) Representative images of intravital two‐photon imaging of bone tissues from a3‐GFP mice during osteoporotic conditions without treatment (*A*) and at 12 hours after treatment with risedronate (*B*), alendronate (*C*), or minodronate (*D*). Green, mature osteoclasts expressing the GFP‐fused V‐ATPase a3 subunit; blue, bone tissues (second harmonic generation; SHG). Scale bar: 50 µm. (*E*) Cell shapes were automatically recognized by the image analysis software, and three distinct areas were defined: the initial time frame (*t* = 0) (A); the final time frame (*t* = 5) (C); and the overlap between the two time frames (B). The cell deformation index was calculated as (A + C)/(A + B), which represents the ratio of the area changed during 5 min divided by that of the previous time frame. (*F*) Cell deformation index of mature osteoclasts during osteoporotic conditions at 12 hours after the administration of risedronate, alendronate, or minodronate. Images were obtained from three independent experiments per group. Data are presented as the mean ± SD. UNT = untreated; RIS = risedronate; ALN = alendronate; MINO = minodronate.

During the process of bone resorption, mature osteoclasts tightly adhere to bone surfaces, proton pumps accumulate along the ruffled border membrane, and then extraordinarily high numbers of protons are secreted. We previously revealed that changes in osteoclast motility play an essential role in osteoclastic acidification.^10^, ^11^ To examine the effects of N‐BPs on osteoclast motility and the localization of proton pumps, we utilized fluorescent reporter mice in which GFP is expressed as a fusion protein with the proton pump, vacuolar type H^+^‐ATPase a3 subunit (a3‐GFP mice). Because the a3 subunit is preferentially and abundantly expressed in mature osteoclasts,^13^, ^14^ a3‐GFP mice are suitable for visualizing mature osteoclast motility and the subcellular distribution of proton pumps in osteoclasts in vivo. Risedronate, alendronate, or minodronate was i.v. injected into osteoporotic a3‐GFP mice. After 12 and 24 hours, the bone tissues of mice were visualized to assess the dynamics of GFP^+^ mature osteoclasts and proton pumps (Figs. 2 and S1). Compared to untreated mice (Fig. 2 *A* and Supplementary Video 2), risedronate increased the morphological changes in mature osteoclasts and changed the localization of proton pumps, representing an inhibition of bone resorption, which was the most dramatic at 12 hours after treatment (Figs. 2 *B*, *E*, *F*, S1*B*, *E*, and Supplementary Video 3). Alendronate also increased osteoclast motility and changed proton pump localization most dramatically at 12 hours after treatment, although its effects were less obvious than those of risedronate (Figs. 2 *C*, *F*, S1*C*, *E*, and Supplementary Video 4). In contrast, minodronate increased the motility of mature osteoclasts at 24 hours after injection (Figs. 2 *D*, *F*, S1*D*, *E*, and Supplementary Video 5).

## Discussion

BPs are well‐established drugs for the treatment of bone destructive disorders. Recent studies have reported that radiolabeled BPs, including risedronate, alendronate, and minodronate, attach to the bone surface within 24 hours after i.v. injection.^5^, ^6^, ^7^ Osteoclasts secrete protons on the mineral surface to resorb bone tissues, resulting in the dissociation of BPs from the bone surface. This is followed by the uptake of BPs by endocytosis in mature osteoclasts. Although numerous studies have reported the pharmacological effects of BPs, it remains unclear when bisphosphonates are actually effective in vivo. In this study, using an intravital bone imaging system with a pH‐sensing chemical fluorescent probe, we analyzed the time‐dependent effects of BPs, and showed that risedronate, alendronate, and minodronate inhibited proton secretion by mature osteoclasts in living bone tissues just 12 hours after a single administration. In addition to inhibiting osteoclastic acidification, risedronate was also the most effective at increasing osteoclast motility and changing the localization of proton pumps, leading to an inhibition of bone resorption within a short period after administration.

BPs have similarities and differences in their mechanisms of action involving their binding affinities with HAP and in their FPPS inhibitory activities.^15^, ^16^ BPs with different binding affinities have different relative distributions in bone and cells. These properties affect the potency and side effects of the drug. For example, risedronate exhibits a distinctive profile of higher enzyme binding and is the strongest inhibitor of FPPS compared to other BPs. In contrast, risedronate has a significantly lower mineral binding affinity than other BPs.^17^ Risedronate is effective on all fracture types, particularly nonvertebral fractures. This effect may be explained by its lower mineral affinity, which may enable a wider distribution within bones. The strong inhibitory action of risedronate at cellular sites may also contribute to its rapid action in fractures.^18^ Although imaging techniques can be applied only to animal models because of technical limitations, our intravital imaging studies may also explain the reasons why different drugs have different efficacies and side effects.

Previous studies using conventional bone histological analyses reported giant mature osteoclasts both in mice and in patients treated with BPs.^19^, ^20^ In accordance with previous results, we observed large and nonmotile multinucleated osteoclasts in bone tissues 24 hours after treatment with BPs, and found that these osteoclasts were not associated with bone resorption activities (Fig. S1). In response to the inhibitory effects of BPs, osteoclasts fuse with each other to increase their activities, suggesting that giant osteoclasts should not be interpreted as a result of increased bone resorption.

In conclusion, our intravital bone imaging techniques could be used to study the in vivo action of antibone resorptive drugs on bone metabolism and provide insights into their effective therapeutic use for a wide range of bone diseases.

## Disclosure

The authors have declared that no conflict of interest exists.

## Supporting information

Supporting Data S1.

Supporting Video S1.

Supporting Video S2.

Supporting Video S3.

Supporting Video S4.

Supporting Video S5.

## References

[1] Russell RG . Bisphosphonates: The first 40 years. Bone. 2011; 49:2–19. 21555003 10.1016/j.bone.2011.04.022

[2] Fisher JE , Rogers MJ , Halasy JM , et al. Alendronate mechanism of action: geranylgeraniol, and intermediate in the mevalonate pathway, prevents inhibition of osteoclast formation, bone resorption, and kinase activation *in vitro* . Proc Natl Acad Sci USA. 1999; 96:133–8. 9874784 10.1073/pnas.96.1.133 PMC15105

[3] Kavanagh KL , Guo K , Dunford JE , et al. The molecular mechanism of nitrogen‐containing bisphosphonates as anti‐osteoporosis drugs. Proc Natl Acad Sci USA. 2006; 103:7829–934. 16684881 10.1073/pnas.0601643103 PMC1472530

[4] Nancollas GH , Tang R , Phipps RJ , et al. Novel insights into actions of bisphosphonates on bone: differences in interactions with hydroxyapatite. Bone. 2006; 38:617–27. 16046206 10.1016/j.bone.2005.05.003

[5] Motaleb MA , Adli AS , El‐Tawoosy M , et al. An easy and effective method for synthesis and radiolabelling of risedronate as a model for bone imaging. J Labelled Comp Radiopharm. 2016; 59:157–63. 26955900 10.1002/jlcr.3384

[6] Masarachia P , Weinreb M , Balena R , et al. Comparison of the distribution of [3H]alendronate and [3H]etidronate in rat and mouse bones. Bone. 1996; 19:281–90. 8873969 10.1016/8756-3282(96)00182-2

[7] Hongo H , Sasaki M , Kobayashi S , et al. Localization of minodronate in mouse femora through isotope microscopy. J Histochem Cytochem. 2016; 64:601–22. 27666429 10.1369/0022155416665577 PMC5037504

[8] Roelofs AJ , Coxon FP , Ebetino FH , et al. Fluorescent risedronate analogues reveal bisphosphonate uptake by bone marrow monocytes and localization around osteocytes *in vivo* . J Bone Miner Res. 2010; 25:606–16. 20422624 10.1359/jbmr.091009 PMC3153397

[9] Roelofs AJ , Stewart CA , Sun S , et al. Influence of bone affinity on the skeletal distribution of fluorescently labeled bisphosphonates *in vivo* . J Bone Miner Res. 2012; 27:835–47. 22228189 10.1002/jbmr.1543

[10] Kikuta J , Wada Y , Kowada T , et al. Dynamic visualization of RANKL and Th17‐mediated osteoclast function. J Clin Invest. 2013; 123:866–73. 23321670 10.1172/JCI65054 PMC3561830

[11] Maeda H , Kowada T , Kikuta J , et al. Real‐time intravital imaging of pH variation associated with osteoclast activity. Nat Chem Biol. 2016; 12:579–85. 27272564 10.1038/nchembio.2096

[12] Sun‐Wada G‐H , Tabata H , Kawamura N , et al. Direct recruitment of H^+^‐ATPase from lysosomes for phagosomal acidification. J Cell Sci. 2009; 122:2504–13. 19549681 10.1242/jcs.050443

[13] Toyomura T , Oka T , Yamaguchi C , et al. Three subunit a isoforms of mouse vacuolar H^+^‐ATPase. Preferential expression of the a3 isoform during osteoclast differentiation. J Biol Chem. 2000; 275:8760–5. 10722719 10.1074/jbc.275.12.8760

[14] Toyomura T , Murata Y , Yamamoto A , et al. From lysosomes to the plasma membrane. J Biol Chem. 2003; 278:22023–30. 12672822 10.1074/jbc.M302436200

[15] Ebetino FH , Hogan A‐ML , Sun S , et al. The relationship between the chemistry and biological activity of the bisphosphonates. Bone. 2011; 49:20–33. 21497677 10.1016/j.bone.2011.03.774

[16] Kavanagh KL , Guo K , Wu X , et al. The molecular mechanism of nitrogen‐containing bisphosphonates as antiosteoporosis drugs. Proc Natl Acad Sci USA. 2006; 103:7829–34. 16684881 10.1073/pnas.0601643103 PMC1472530

[17] Russell RG , Watts NB , Ebetino FH , et al. Mechanisms of action of bisphosphonates: similarities and differences and their potential influence on clinical efficacy. Osteoporos Int. 2008; 19:733–59. 18214569 10.1007/s00198-007-0540-8

[18] Chen LX , Zhou ZR , Li YL , et al. Comparison of bone mineral density in lumbar spine and fracture rate among eight drugs in treatments of osteoporosis in men: a network meta‐analysis. PLoS One. 2015; 10:e0128032. 26010450 10.1371/journal.pone.0128032 PMC4444106

[19] Matsumoto T , Nagase Y , Iwasawa M , et al. Distinguishing the proapoptotic and antiresorptive functions of risedronate in murine osteoclasts: role of the Akt pathway and the ERK/Bim axis. Arthritis Rheum. 2011; 63:3908–17. 21898348 10.1002/art.30646

[20] Mac‐Way F , Trombetti A , Noel C , et al. Giant osteoclasts in patients under bisphosphonates. BMC Clin Pathol. 2014; 8:14–31. 10.1186/1472-6890-14-31 PMC4094788 25024641

