# Supplementary material for: Dynamic Analyses of the Short‐Term Effects of Different Bisphosphonates Using Intravital Two‐Photon Microscopy
Source: JBMR Plus. 2018 Jun 22;2(6):362–6. doi: 10.1002/jbm4.10057 (PMC6237210; doi:10.1002/jbm4.10057)
Supplement: Supplementary file 1 — Supporting Data S1. [file JBM4-2-362-s001.docx]

**Supplementary Figure**

**
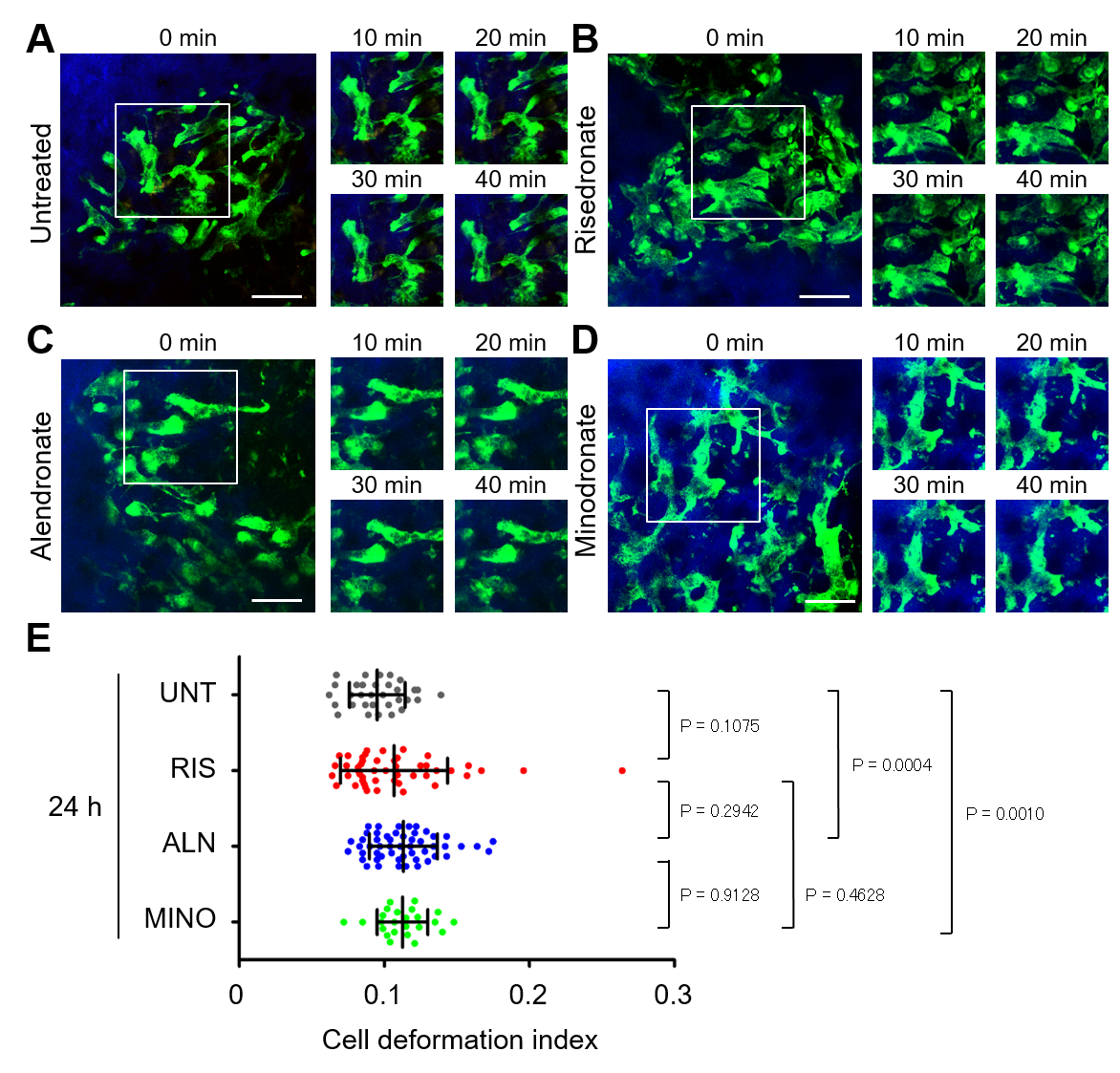
**

**Supplementary Figure 1. Effects of bisphosphonates on osteoclast motility 24 h after treatment. A–D,** Representative images of intravital two-photon imaging of bone tissues from a3-GFP mice during osteoporotic conditions without treatment (**A**) and at 24 h after treatment with risedronate (**B**), alendronate (**C**), or minodronate (**D**). Green, mature osteoclasts expressing the GFP-fused V-ATPase a3 subunit; blue, bone tissues (second harmonic generation, SHG). Scale bar: 50 μm. **E,** Cell deformation index of mature osteoclasts during osteoporotic conditions at 24 h after the administration of risedronate, alendronate, or minodronate. Images were obtained from three independent experiments per group. Data are presented as the mean ± SD.

**Supplementary Videos**

**Supplementary Video 1.** Intravital two-photon imaging of osteoclastic acidification in skull bone tissues from TRAP-tdTomato mice during osteoporotic conditions at 12 h after single intravenous administration of risedronate, alendronate or minodronate. Mice were treated with pHocas-3 via subcutaneous injection over 3 days. Green, fluorescent signals from pHocas-3; red, mature osteoclasts expressing TRAP-tdTomato. Scale bar: 50 µm. Playback speed, 900×.

**Supplementary Video 2.** Intravital two-photon imaging of osteoclast motility in skull bone tissues from a3-GFP mice during osteoporotic conditions without BP treatment. Green, mature osteoclasts expressing a3-subunit-fused GFP. Scale bar: 50 µm. Playback speed, 300×.

**Supplementary Video 3.** Intravital two-photon imaging of osteoclast motility in skull bone tissues from a3-GFP mice during osteoporotic conditions at 12 h after single intravenous administration of risedronate. Green, mature osteoclasts expressing a3-subunit-fused GFP. Scale bar: 50 µm. Playback speed, 300×.

**Supplementary Video 4.** Intravital two-photon imaging of osteoclast motility in skull bone tissues from a3-GFP mice during osteoporotic conditions at 12 h after single intravenous administration of alendronate. Green, mature osteoclasts expressing a3-subunit-fused GFP. Scale bar: 50 µm. Playback speed, 300×.

**Supplementary Video 5.** Intravital two-photon imaging of osteoclast motility in skull bone tissues from a3-GFP mice during osteoporotic conditions at 12 h after single intravenous administration of minodronate. Green, mature osteoclasts expressing a3-subunit-fused GFP. Scale bar: 50 µm. Playback speed, 300×.
